# Intention-to-treat outcomes utilising a stringent event definition in children and young people treated with tisagenlecleucel for r/r ALL through a national access scheme

**DOI:** 10.1038/s41408-024-01038-2

**Published:** 2024-04-15

**Authors:** Macarena Oporto Espuelas, Saskia Burridge, Amy A. Kirkwood, Denise Bonney, Kelly Watts, Geoff Shenton, Katarzyna A. Jalowiec, Maeve A. O’Reilly, Claire Roddie, Anna Castleton, Katherine Clesham, Emma Nicholson, Rajesh Alajangi, Shilpa Prabhu, Lindsay George, Ben Uttenthal, Maria Gabelli, Lorna Neill, Caroline Besley, Sridhar Chaganti, Robert F. Wynn, Jack Bartram, Robert Chiesa, Giovanna Lucchini, Vesna Pavasovic, Anupama Rao, Kanchan Rao, Juliana Silva, Sujith Samarasinghe, Ajay Vora, Peter Clark, Michelle Cummins, David I. Marks, Persis Amrolia, Rachael Hough, Sara Ghorashian

**Affiliations:** 1 Infection, Immunity and Inflammation, UCL Great Ormond Ormond Street Institute of Child Health, London, UK; 2 Department of Haematology, Great Ormond Street Hospital, London, UK; 3 Cancer Research UK & Cancer Trials Centre, UCL, London, UK; 4 Department of Blood and Bone Marrow Transplant, Royal Manchester Children’s Hospital, Manchester, UK; 5 Great North Children’s Hospital, Newcastle upon Tyne, UK; 6 Department of Haematology, University College London Hospitals NHS Foundation Trust, London, UK; 7 Department of Haematology, The Christie Hospital NHS Foundation Trust, Manchester, UK; 8 Department of Haematology/Bone Marrow Transplantation, The Royal Marsden NHS Foundation Trust, London, UK; 9 Institute of Cancer Research, London, UK; 10 Department of Haematology/Bone Marrow Transplant, University Hospitals Bristol and Weston NHS Foundation Trust, Bristol, UK; 11 Centre for Clinical Haematology, Queen Elizabeth Hospital, Birmingham, UK; 12 Cambridge University Hospital NHS Foundation Trust, Cambridge, UK; 13 Department of Bone Marrow Transplant, Great Ormond Street Hospital, London, UK; 14 Pediatric Onco-hematology and Hematopoietic Stem Cell Transplantation, Woman and Child Health Department, University of Padova, Padua, Italy; 15 NHS England, London, UK; 16 Bristol Royal Hospital for Children, Bristol, UK; 17 Department of Haematology, University Hospitals Bristol, Bristol, UK; 18 Developmental Biology and Cancer, UCL Great Ormond Ormond Street Institute of Child Health, London, UK

**Keywords:** Acute lymphocytic leukaemia, Paediatrics

## Abstract

CAR T-cell therapy has transformed relapsed/refractory (r/r) B-cell precursor acute lymphoblastic leukaemia (B-ALL) management and outcomes, but following CAR T infusion, interventions are often needed. In a UK multicentre study, we retrospectively evaluated tisagenlecleucel outcomes in all eligible patients, analysing overall survival (OS) and event-free survival (EFS) with standard and stringent definitions, the latter including measurable residual disease (MRD) emergence and further anti-leukaemic therapy. Both intention-to-treat and infused cohorts were considered. We collected data on feasibility of delivery, manufacture, toxicity, cause of therapy failure and followed patients until death from any cause. Of 142 eligible patients, 125 received tisagenlecleucel, 115/125 (92%) achieved complete remission (CR/CRi). Severe cytokine release syndrome and neurotoxicity occurred in 16/123 (13%) and 10/123 (8.1%), procedural mortality was 3/126 (2.4%). The 2-year intent to treat OS and EFS were 65.2% (95%CI 57.2–74.2%) and 46.5% (95%CI 37.6–57.6%), 2-year intent to treat stringent EFS was 35.6% (95%CI 28.1–44.9%). Median OS was not reached. Sixty-two responding patients experienced CAR T failure by the stringent event definition. Post failure, 1-year OS and standard EFS were 61.2% (95%CI 49.3–75.8) and 55.3% (95%CI 43.6–70.2). Investigation of CAR T-cell therapy for B-ALL delivered on a country-wide basis, including following patients beyond therapy failure, provides clinicians with robust outcome measures. Previously, outcomes post CAR T-cell therapy failure were under-reported. Our data show that patients can be successfully salvaged in this context with good short-term survival.

## Introduction

B-cell precursor acute lymphoblastic leukaemia (B-ALL) is the most common form of leukaemia in children [1]. Improvements in risk-stratified anti-leukaemic treatment and supportive care have led to an overall survival (OS) in this disease approaching 95% in children and 75% in young adults [2, 3]. However, around 20% of patients relapse. Since the introduction of anti-CD19 directed chimeric antigen receptor T-cell therapies (CAR T-cell) a significant proportion of patients previously deemed incurable can obtain long-term remissions. The ELIANA study demonstrated OS of 63% and event-free survival (EFS) of 44% three years following tisagenlecleucel CAR T-cell therapy [4]. Since regulatory approval, a growing number of real-world cohorts have reported results generally consistent with those of ELIANA [5]. However, the EFS definition utilised in ELIANA did not include all events such as early B-cell recovery or emergence of measurable residual disease (MRD), both of which may require further intervention. Thus, the true proportion of patients potentially cured by tisagenlecleucel as a stand-alone therapy is not known. Additionally, these cohorts have considered only infused patients [6–9], or successfully apheresed patients [10] – thereby limiting applicability.

We present here a comprehensive, retrospective, multi-centre, national report of all patients eligible to receive tisagenlecleucel within the UK National Health Service (NHS) from allocation to CAR T-cell treatment through to post-infusion outcomes. For the first time, we can accurately report true intention-to-treat outcomes of patients being treated with tisagenlecleucel even after failure of this therapeutic. Notably, we provide a stringent measure of EFS (stringent EFS) that includes all events relevant to patients and clinicians [11], which reflects the true proportion of patients rendered disease-free by this therapeutic agent alone. We detail therapeutic strategies given after CAR T and patient outcomes following these. We believe these data will provide clinicians with robust information truly applicable to patients at the point of screening for CAR T-cell therapy.

## Methods

### Study design and participants

We carried out a retrospective, multi-centre study across the UK. Potential CAR T candidates are discussed at a fortnightly national CAR T ALL panel, where eligibility for tisagenlecleucel is assessed based on licensed indications: refractory disease, second (or later) or post haematopoietic stem cell transplantation (HSCT) relapse. Eligible patients formed the intention-to-treat (ITT) cohort and patients who received tisagenlecleucel formed the infused cohort. We included patients deemed eligible for tisagenlecleucel from Dec 1, 2018 to June 15, 2022. Consent for deidentified or pseudo-anonymised data collection and sharing was obtained from patients/parents as per institutional guidance.

### Procedures

Patients were identified using the contemporaneously written minutes of all national CAR T-cell panel meetings. Data were retrospectively collected on standardised datasheets distributed to all UK CAR T treating centres, extracted from local medical patient records by physicians or nurse practitioners and centrally reviewed and analysed. Patients underwent a single CAR T-cell infusion preceded by lymphodepletion and were followed according to local practice with a data cut-off of March 20, 2023. Bone marrow (BM) disease burden was based on molecular MRD results from quantitative PCR of immunoglobulin H or T-cell receptor (IgH/TCR) rearrangements, flow cytometry or BM morphology in decreasing order of priority (morphology was only considered for patients with >50% disease). Cytogenetic risk classification was based on the UKALL cytogenetic risk categories [12]. Blinatumomab response prior to tisagenlecleucel was defined as a binary variable, complete response with or without haematologic recovery (CR/CRi) or non-response as previously defined [13, 14]. CR/CRi and relapse were defined as per international consensus criteria [13].

### Outcomes

OS was defined as time from allocation to CAR T (ITT cohort) or time of infusion (infused cohort) to death of any cause. EFS was defined as time from allocation (ITT)/infusion (infused) to failure to respond, frank relapse after having responded - or death. Failure to respond was considered to occur on the day following tisagenlecleucel infusion (but was confirmed by disease assessment on day 30). Additionally, we analysed stringent EFS which encompassed all clinically-relevant events after CAR T infusion including death, relapse, measurable residual disease (MRD) emergence or initiation of further anti-leukaemic treatment due to early loss of B-cell aplasia (LBCA) with undetectable disease. MRD emergence was defined as BM disease with less than 1% malignant cells determined by flow cytometry or quantitative IgH PCR within the quantitative range for the relevant assay [11]. B-cell aplasia as a surrogate marker of CAR T-cell persistence was defined as <10 CD19+ cells/μl by flow cytometry in the bone marrow/peripheral blood. Duration of B-cell aplasia was defined as time from infusion until loss of B-cell aplasia or CD19 positive relapse, competing risks were non-response, disease re-emergence other than CD19 positive and death. We also provide a detailed analysis of post-infusion outcomes for responding patients who subsequently failed CAR T-cell therapy by a stringent event definition. For these, survival was defined as time from stringent event until death (OS) or until relapse or death (EFS). In time to event analyses, patients without an event were censored at last follow-up, except EFS after CAR T infusion/allocation where patients were censored at further therapy where applicable [15]. We analysed the cumulative incidence of relapse with failure to respond or death in remission as competing risks. Similarly, failure to respond, death in remission, unknown relapse phenotype, myeloid escape and CD19 positive/negative relapses were competing events for each other. In the analysis of cumulative incidence of relapse, all relapse events after CAR T were captured, including if they occurred after other post-tisagenlecleucel therapy.

Cytokine release syndrome (CRS) and immune effector cell-associated neurological syndrome (ICANS) were graded according to ASTCT consensus criteria [16]. Prior to their publication, CRS and ICANS were graded according to NIH criteria [17] and CTCAE criteria respectively. CTCAE (version 5.0) criteria were used to grade infection, prolonged cytopenias and hypogammaglobulinemia. Hypogammaglobulinemia was defined as IgG <5 g/L and immunoglobulin replacement was initiated following local protocols.

Procedural mortality was defined as death not attributable to disease following lymphodepletion and within 2 months after infusion.

### Statistical analysis

Baseline features as well as incidence of toxicities were descriptively analysed. The Kaplan-Meier method was used for all survival analyses, curves were compared with the log-rank test. Median follow-up times were calculated with the reverse Kaplan-Meier method. We constructed Cox proportional hazard models for univariable and multivariable analysis of baseline characteristics/risk factors influencing survival outcomes. The Bayesian information criterion guided choice of the final multivariable model, together with prior evidence and clinical significance of predictors. We calculated the cumulative incidence of relapse considering competing risks (defined in Supplementary methods). For survival after CAR T failure, HSCT post-CAR was analysed as a time-varying-covariate (TVC) [18]. All statistical analyses were carried out with R (version 4.2.3).

## Results

### Study population

**Fig. 1:**
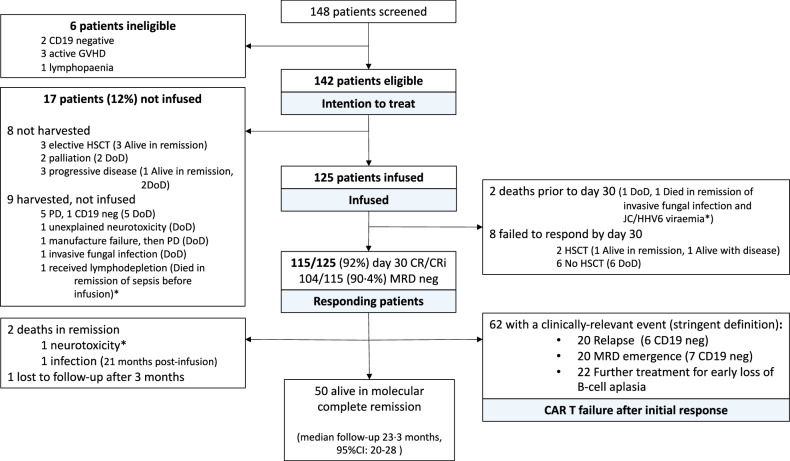
Patient flowchart. CR/CRi complete remission with/without haematological recovery, DoD died of disease, PD progressive disease, HSCT allogeneic haematopoietic stem cell transplantation, MRD minimal residual disease. *Patients died from procedural mortality (*n* = 3).

148 patients were screened (Fig. 1), 6 were deemed ineligible for tisagenlecleucel due to active graft-versus-host disease (*n* = 3), CD19 negative disease (2), and lymphopenia precluding leukapheresis (1). The intention-to-treat cohort was therefore formed by 142 eligible patients. Overall, 17 eligible patients (12%) were not infused. Eight patients were not harvested: 5 patients underwent alternative therapy or palliation, and 3 had progressive disease. Nine harvested patients were not infused—in 6 this was due to progressive leukaemia, 1 patient succumbed to infection after lymphodepletion (Fig. 1).

Table 1 shows patient baseline characteristics. The median age at CAR T-cell screening was 11 years (IQR 6.5–15.9, range 0.7–25.7), 79/142 (55.6%) were male. Fifteen patients (10.6%) were infants at diagnosis, and 14 of these had KMT2A rearrangement. 44/142 (31.0%) presented with high-risk features and 2 presented as B-cell lymphoblastic lymphoma. Half of the population (74/142, 52.1%) were deemed refractory at some point prior to infusion with tisagenlecleucel. This was a heavily pre-treated cohort with a median of 3 (IQR 2–3) lines prior to CAR T (excluding HSCT) and 58 (40.8%) having received HSCT (one patient had received 2 allografts). 43/142 (30.3%) had received blinatumomab (12/43, 27.9% were non-responders) and 18/142 (12.7%) had received inotuzumab prior to tisagenlecleucel. Six patients (4.2%) had received experimental anti-CD19 and/or CD22 directed CAR T-cell products prior to consideration for tisagenlecleucel. Sixty-four patients (45.1%) had central nervous system (CNS) involvement prior to CAR T referral and 17 (12%) non-CNS extramedullary disease (EMD), with testicular disease being the single most common site (Supplementary Table 1).

**Table 1:** Baseline characteristics.

	*n* = 142		
Variable	*n*	%	Range
	or median	or IQR	
Male	79	55.6%	
Age (at screening)	11 years	6.5–15.9	0.7–25.7
Age (at infusion)	11.7 years	6.9–16	0.9–26.3
Infants at diagnosis	15	10.6%	
(age ≤12 months)			
White cell count at diagnosis			
(cells 10^9^/L)			
Median	40	10–154	0.8–171
Low (<50)	61	43%	
High (>=50)	48	33.8%	
CNS/extramedullary disease			
CNS at diagnosis	19	13.4%	
CNS at relapse	52	36.6%	
CNS at any point	64	45.1%	
Non-CNS EM relapses (both isolated and combined)	17	12.00%	
Isolated non-CNS EM relapses	7	4.9%	
Cytogenetic risk			
Good risk	35	24.6%	
ETV6-RUNX1	18	12.7%	
High hyperdiploid	17	12.0%	
Intermediate risk	53	37.3%	
t(1;19)/TCF3-PBX1	2	1.4%	
B other	51		
High risk	44	31.0%	
KMT2A rearranged*	21	14.8%	
Ph+	11	7.7%	
iAMP21	6	4.2%	
Low hypodiploidy (30 -39 chr) or near haploidy (24-29 chr)	2	1.4%	
t(17;19)/ TCF3-HLF	2	1.4%	
High risk cytogenetics not further specified	2	1.4%	
Unknown	10	7.0%	
Indication for CART			
Primary refractory	7	4.9%	
Relapse	132	93.0%	
Refractory status			
At any timepoint	74	52.1%	
Primary refractory	33	23.2%	
Refractory relapse	50	35.2%	
N of relapses	2	1–2	1–5
N therapy lines (excluding HSCT)	3	2–3	1–8
Prior HSCT	58	40.8%	
Blinatumomab exposure	43	30.3%	
Inotuzumab exposure	18	12.7%	

*Further details in Supplementary Table 2.

Disease status was assessed prior to lymphodepletion (Table 2). Patients had a median BM disease burden of 2% (IQR 0.09–12%) blasts. 57/125 of infused patients (45.6%) had low (<5% blasts) BM disease burden, 48/125 (38.4%) proceeded with high (≥5%) disease burden and 15/125 (12%) had undetectable BM disease. There were no patients with CNS3 disease. Three patients with EMD proceeded with either stable (*n* = 2) or reduced (*n* = 1) disease burden compared with previous MRI/US imaging.

**Table 2:** Disease status prior to lymphodepletion.

	*n* = 125 infused patients	
	*n*	%/IQR
BM status		
MRD negative	15	12.0%
<5%	57	45.6%
≥5%	48	38.4%
Unknown	5	4.0%
BM disease burden	2%	0.09–12%,
		range 0–100%
CNS status		
CNS1	103	82.4%
CNS2	3	2.4%
CNS3	–	–
Unknown	19	15.2%
Non-CNS EMD	3*	2.4%

*3 patients with EMD had “stable” finding on MRI/US imaging, 1 patient “reduced” compared with previous. None had signs of progression or activity prior to lymphodepletion.

All patients received lymphodepletion comprising intravenous fludarabine (4 days at 30 mg/m^2^/day) and cyclophosphamide (2 days at 500 mg/m^2^/day). In 3 patients fludarabine doses were adjusted to renal function.

### Leukapheresis and manufacture

134 patients underwent harvest with a median of 1 day needed for leukapheresis (IQR 1–1; range 1–4) (Table 3). In one case, the leukapheresis product did not meet manufacturer’s guidance (low CD3 count) but manufacture was ultimately successful. In 5 cases (3.7%) the manufactured product did not meet manufacturer’s release criteria: 1 was infused as an out-of-specification product (viability <70%), 3 underwent successful repeat harvest and subsequent manufacture. One patient progressed before another harvest could be re-attempted (Supplementary Table 3).

**Table 3:** Leukapheresis and manufacture details (*n* = 134 patients with attempted harvest).

	Median/n	IQR/%
CD3 collected (x 10^9^)	2.6	1.2–4.1
CD45 or TNC collected (x 10^9^)	5.6	3.1–8.4
Satisfactory apheresis	134	100%
Apheresis days	1	1–1,
		range 1–4
Satisfactory manufacture	128	95.5%
Manufacture failure	5*	3.7%
CAR T dose (10^6^/kg) *n* = 112	2.8	2.0–3.5
CAR T dose (x 10^8^) *n* = 13	1.5	1.4–1.6
CAR T-cell transduction efficiency %	17.4%	12–23.1%
Time from screening to infusion (days)	60	50–80

*Details of manufacture failure patients and their outcomes can be found in Supplementary Table 3.

*TNC* total nucleated cells.

### Outcomes

Outcomes for the 17 non-infused patients are shown in Fig. 1. Median survival for the 17 non-infused patients was 4.4 months (95%CI 3, - not reached [NR]), 4/17 were alive in remission at last follow-up.

The CR/CRi rate at day 30 for the 125 infused patients was 92% (115/125). Of 115 responders, 104 (90.4%) were negative for BM MRD by IgH/TCR PCR. Two patients died prior to day 30 and were not evaluable for response: one died of refractory disease five days after infusion, the other died of fungal pneumonia whilst in remission on day 24. Of 123 evaluable patients at day 30, 8 did not achieve CR/CRi: 7 died of disease (all within 16 months) and 1 is alive in remission 2 years after HSCT.

**Fig. 2:**
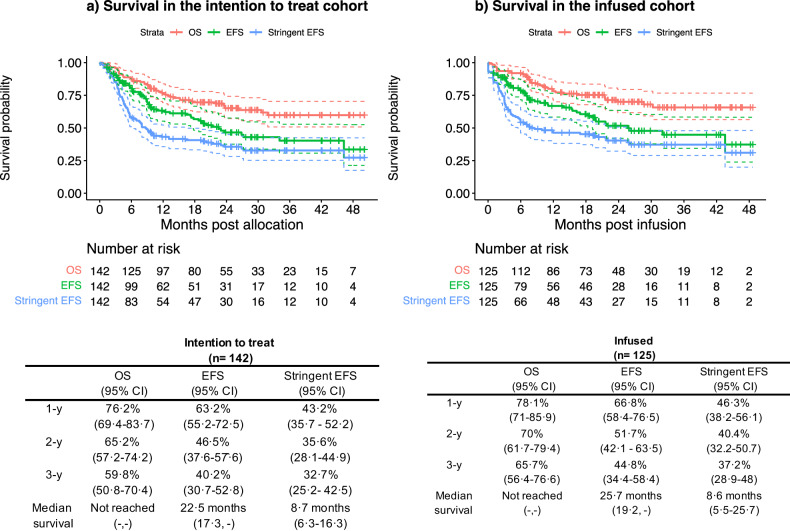
Survival outcomes after tisagenlecleucel in r/r B-ALL. Survival in the intention to treat (**a**) and infused (**b**) cohorts. OS overall survival, EFS event-free survival as per ELIANA definition, stringent EFS stringent event-free survival.

Survival for both the ITT and infused cohorts are shown in Fig. 2a, b. With a median follow-up of 26.3 months (IQR 19.7–36.7), the 2-year OS in the ITT cohort was 65.2% (95%CI 57.2–74.2%) and in the infused cohort was 70% (95%CI 61.7–79.4%). Two-year EFS as per ELIANA criteria was 46.5% (95%CI 37.6–57.6%) and 51.7% (95%CI 42.1–63.5%) for the ITT and infused cohorts respectively. Two-year stringent EFS was 35.6% (95%CI 28.1–44.9%) for the intention-to-treat cohort and 40.4% (95%CI 32.2–50.7%) for the infused cohort. The median OS for the ITT cohort was not reached, median EFS as per ELIANA criteria was 22.5 months (95%CI 17.3—NR), median stringent EFS was 8.7 months (95%CI 6.3–16.3). In the infused population the median OS was not reached, the median EFS was 25.7 (19.2-NR) months and the median stringent EFS was 8.6 months (5.5–25).

Using the standard definition we noted a worse EFS in association with greater disease burden (≥5%, HR 1.95, *p* = 0.02), prior inotuzumab exposure (HR 2.21, *p* = 0.03), refractory disease, particularly refractory relapse (HR 3.05, *p* < 0.0001), blinatumomab non-response (HR 2.90, *p* = 0.02) (Table 4) and number of therapy lines by univariable regression analysis (Supplementary Fig. 1). However, follow-up for the 18 patients who had received inotuzumab (Supplementary Fig. 1) was short and as such, its longer-term effect could not be reliably estimated. We tested these and other clinically relevant variables in multivariable models. In the final adjusted model only refractory disease (HR 2.33, 95%CI 1.1–4.9), *(p* = 0.02) was noted to be significantly associated with worse EFS after tisagenlecleucel. In contrast to frontline chemotherapy [19], age at tisagenlecleucel screening did not impact survival (Table 4).

**Table 4:** Impact of baseline and other characteristics on event-free survival (as per ELIANA definition) in the intention to treat cohort.

			Univariable regression analysis		Multivariable regression analysis	
Variable	Levels	Events/N	HR (95%CI)	*p* value	HR (95%CI)	*p* value
Cytogenetic risk	Good	13/35	1	0.64	1	0.48
	Intermediate	25/53	1.37 (0.7–2.7)		1.60 (0.7–3.7)	
	Poor	20/44	1.22 (0.6–2.5)		1.64 (0.6–4.1)	
Infant	Infant	5/15	0.77 (0.3, 1.9)	0.57	–	–
Age at diagnosis (years)		61/136	1.04 (1–1.1)	0.06	–	–
Age at CAR T screening (years)		64/142	1.01 (1–1.1.1)	0.63	–	–
CNS involvement*	Yes	27/64	0.90 (0.5–1.5)	0.68	–	–
Non-CNS EMD*	Yes	10/17	1.73 (0.9–3.4)	0.14	–	–
Prior SCT	Yes	26/58	0.66 (0.4–1.1)	0.11	0.79 (0.4–1.5)	0.50
Refractory at any point	Yes	39/74	1.82 (1.1–3.1)	0.02	2.34 (1.1–4.9)	0.02
Primary refractory disease	Yes	16/33	1.03 (0.6–1.9)	0.91	–	–
Refractory at relapse	Yes	30/50	3.05 (1.7–5.3)	<0.0001	–	–
Number of relapses		63/139	1.07 (0.8–1.4)	0.66	–	–
Number of therapy lines excluding SCT		62/138	1.30 (1.0–1.6)	0.03	1.29 (0.9–1.8)	0.15
Prior Blinatumomab	Yes	18/43	1.00 (0.6–1.7)	0.99	–	–
Blinatumomab response	Blinatumomab naïve	46/97	1	0.02	1	0.13
	Responder	10/31	0.66 (0.3–1.3)		0.43 (0.2–1.2)	
	Non responder	8/12	2.90 (1.4–6.2)		1.25 (0.3–4.6)	–
Prior Inotuzumab	Yes	11/18	2.21 (1.2–4.3)	0.03	2.2 (0.9–5.4)	0.11
BM disease burden prior to lymphodepletion	High (≥5%)	26/49	1.95 (1.1–3.4)	0.02	1.60 (0.8–3.1)	0.16
CAR T dose (10^6^/kg)		48/115	1.00 (1.0–1.1)	0.67	–	–
Severe CRS	Yes	8/16	1.54 (0.7–3.3)	0.29	–	–

*At any timepoint before CART.

### Toxicity

Toxicity data were available for 123 of 125 infused patients. Tisagenlecleucel was generally well tolerated, the procedural mortality was 2.4% (accounting for 3/126 patients who proceeded with lymphodepletion) (Fig. 1). Although 106/123 (86.2%) patients suffered cytokine release syndrome (CRS), this was severe (grade 3–4) in only 16/123 (13%), with a median duration of 3 days (Table 5a). Immune effector cell associated neurotoxicity syndrome (ICANS) affected 26/123 (21.1%) patients and was severe in 10 (8.1%), the median duration was 3 days. One patient died of encephalopathy on day 51 post-infusion, an extensive infectious screen was negative.

**Table 5:** **a** Toxicity: CRS and ICANS. **b** Toxicity: Cytopenia beyond day 30 after CAR T infusion. **c** Toxicity: infection.

a		
	*n* = 123	
	CRS	ICANS
	*n* (%)	*n* (%)
Occurrence	106 (86.2%)	26 (21.1%)
(Grade 1–5)		
Max. grade		
0	17 (13.8%)	97 (78.9%)
1	52 (42.3%)	11 (8.9%)
2	38 (30.9%)	5 (4.1%)
3	11 (8.9%)	6 (4.9%)
4	5 (4.1%)	3 (2.4%)
5	–	1 (0.8%)
Duration(days)	3 (2–5.8)	3 (2–4)
Treatment for CRS/ICANS		
ITU	29 (23.6%)	
ITU duration (days)	3 (2–4.1)	
Tocilizumab	53 (43.1%)	
Anakinra	6 (4.9%)	
Steroids	20 (16.3%)	
Siltuximab	1 (0.8%)	

b	
*n* = 95 (of 115 responding patients)	
Any grade of cytopenia	56 (58.9%)
Max grade	
0	34 (35.8%)
1	6 (6.3%)
2	5 (5.3%)
3	13 (13.7%)
4	33 (34.7%)
5	–
Cytopenia, grade unconfirmed	4 (4.2%)
Lineage affected	
N + T + A	12 (12.6%)
N + T	14 (14.7%)
N + A	2 (2.1%)
N only	23 (24.2%)
A only	5 (5.3%)
T only	2 (2.1%)
Unknown	37 (38.9%)
Supportive care*	
GCSF	29 (30.5%)
Transfusions (platelets/blood)	10 (10.5%)
Anti-fungal prophylaxis	18 (18.9%)
Antibiotic prophylaxis	8 (8.4%)
Not reported	50 (49.5%)

c	
*n* = 123 patients, 45 episodes of infection	
(median 1 episode/patient, IQR 1–1.5)	
	n (% of patients)
Any infection	35 (28.5%)
Type*	
Viral infection	11 (8.9%)
Sepsis/bacteriaemia	11 (8.9%)
Other	10 (8.1%)
Febrile neutropenia	9 (7.3%)
Invasive fungal infection	4 (3.2%)
Max. grade	
0	88 (71.5%)
1	1 (0.81%)
2	17 (13.8%)
3	23 (18.7%)
4	3 (2.4%)
5	1 (0.81%)

*Some patients received several of these interventions.

*9 patients suffered 2 and 1 patient 3 infection episodes.

*CRS* cytokine release syndrome, *ICANS* immune-effector cell associated neurologic syndrome, *ITU* intensive treatment unit hospitalization, *N* neutropenia, *T* thrombocytopenia, *A* anaemia, *GCSF* granulocyte colonies stimulating factor.

Cytopenias persisting or occurring beyond day 30 post-infusion were noted in 56 of 95 (58.9%) evaluable patients (Table 5b), median duration was 57.5 days (IQR 20–90) after day 30 and 33/95 (34.7%) were grade 4. Neutropenia was the commonest cytopenia, with 23/95 (24.2%) having isolated neutropenia and 28/95 patients (29.5%) having neutropenia in combination with anaemia/thrombocytopenia. Supportive interventions for cytopenia are shown in Table 5b.

There were 45 episodes of infection in 35/123 (28.5%) evaluable patients, 26/123 (21.1%) had 1, 8 (6.5%) had 2 and 1 (0.8%) had 3 episodes of infection; 27/45 (60%) of infections were severe, i.e., grade 3 or higher (Table 5c). One patient died of a combined JC/HHV6 CNS infection and invasive fungal chest infection (see procedural mortality above). The most frequent infections were viral infections (11/123, 8.9%, Supplementary Table 4) which were generally mild (9/11, 81.8% grade 1–2) and sepsis/bacteriaemia (11/123, 8.9%), all of which were severe (10/11 grade 3 and 1/11 grade 4). Other type of infections (10/123, 8.1%) included skin/deep tissue infections (3), respiratory infections (2), urinary tract infections (2), otitis media (1), conjunctivitis (1) and pancreatitis (1). Febrile neutropenia (grade 3) affected 9/123 (7.3%); all concomitantly affected by CRS, which may have been a confounder.

**Fig. 3:**
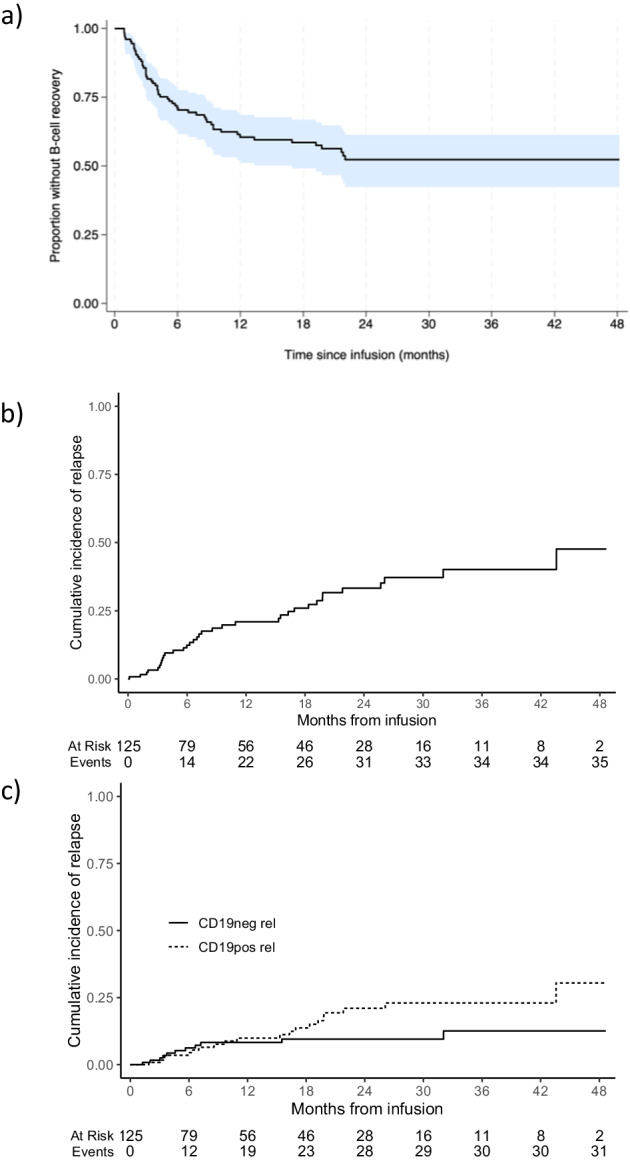
Further outcomes for infused cohort (*n* = 125). **a** Probability of remaining in B-cell aplasia. Main events are B-cell recovery or CD19 positive relapse, competing events are non-response, disease emergence other than CD19 positive and death. **b** Cumulative incidence of relapse. Death in remission and non-response are competing events. All documented relapses were considered, i.e. including those occurring after MRD emergence and/or other therapy following CAR T. This is because censoring further treatments received after CAR T would underestimate the true incidence of relapse after tisagenlecleucel infusion. **c** Cumulative incidence of relapse stratified by immunophenotype. Competing events non-response, death in remission, myeloid switch and relapse with unknown immunophenotype.

Hypogammaglobulinemia is a predictable consequence of B-cell aplasia induced by CD19 CAR therapy in children, we documented this in 91/104 (87.5%) responders for whom this information was available. Intravenous immunoglobulin (Ig) replacement was started in 58/91 (63.7%) patients by the time of the data cut-off; at later time-points post CAR T infusion, subcutaneous formulations were used. The probability of remaining in B-cell aplasia after tisagenlecleucel infusion is shown in Fig. 3a. For the 55 patients who did have B-cell recovery or CD19 positive MRD re-emergence/relapse, the median time until these events was 4.1 months (IQR 2.4–8.7).

### Cumulative incidence of relapse

The cumulative incidence of frank relapse 1- and 2-years post-infusion was 21% (95%CI: 14–29%) and 33% (95%CI 24–43%) respectively (Fig. 3b). Relapses before further therapeutic intervention occurred between 1.3 and 43.6 months post-infusion. The cumulative incidence of CD19 negative relapse was 8.8% (95%CI 4.4–15) and of CD19 positive relapse 18% (95%CI 11–27) at 2 years post-infusion (Fig. 3c). Three patients experienced myeloid switch leukaemia after tisagenlecleucel: one non-responder (40 days post-infusion) and two responders (98 and 784 days post-infusion), two died of disease; a patient with late myeloid switch was alive with disease at last follow-up. A fourth patient evolved to a myeloid switch 1 month after starting blinatumomab for a CD19 positive MRD relapse following infusion and died of disease. Two of these patients with myeloid switch disease had KMT2A-rearranged disease, making the rate of lineage switch in this subgroup 2 of 18 infused cases (11.1%).

### Outcomes after CAR T-cell failure

**Fig. 4:**
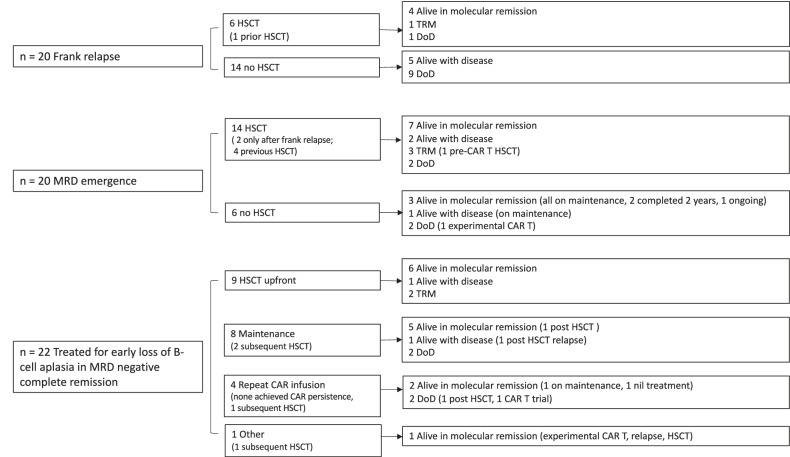
Detailed outcomes after CAR T failure. HSCT allogeneic haematopoietic stem cell transplantation, MRD minimal residual disease, TRM transplant related mortality, DoD died of disease.

All 115 responding patients were evaluable for longer term outcomes (Fig. 1, Responding patients’ box). Two patients died in remission after day 30, one of neurotoxicity (see Toxicity above) and one of late infection. With a median follow-up of 23.3 months (IQR 14.9–32.8), 50 patients were alive and event-free by the stringent definition at the time of data cut-off, a single patient was lost to follow-up (3 months post-infusion). Of the remaining 62 patients (Fig. 1), in whom the median follow-up from infusion was 28.7 months (IQR 20.5–36.3), 20 patients first presented with a frank relapse, 20 patients had emergence of MRD, and 22 patients received further anti-leukaemic therapy for early LBCA with undetectable disease. Outcomes for these 62 patients are detailed in Fig. 4.

Twenty patients relapsed at a median of 7.1 months (95%CI: 4.6–19.8) post tisagenlecleucel. Twelve (60%) had a CD19 positive relapse, 6 (30%) had a CD19 negative relapse, 1 (5%) patient relapsed with myeloid switch leukaemia and in 1, the immunophenotype was unknown. Thirteen (65%) had isolated BM relapse, 3 (15%) BM + non-CNS EMD (EMD sites: 2 orbital, 1 bilateral kidney involvement), 3 (15%) isolated CNS and 1 (5%) isolated EM (maxilla and bilateral kidney lesions) relapse. Six patients underwent HSCT: 4 are alive in molecular remission, 1 died of TRM and 1 died of disease after a further post-HSCT relapse. Of the 14 patients who did not undergo transplant, 5 are alive with disease (follow-up range 0–26.8 months) and 9 died of disease.

Re-emergence of MRD occurred at a median 3 months after infusion (95%CI 2–6). 3/20 (15%) patients never achieved a molecular remission and had rising levels of MRD. 9/20 (45%) had CD19 positive and 7/20 (35%) CD19 negative disease and in the rest (4/20, 20%) the immunophenotype of disease was unknown. 14/20 (70%) patients received HSCT (in 2 cases following a frank relapse), of which 3 died in remission of TRM and 2 died of disease. Within the 6 non-transplanted patients, 4 are alive after starting maintenance treatment: 3 are alive in molecular remission (2 have completed 2 years of maintenance) and 1 is alive with disease.

Of the 22 patients who were treated for early LBCA, LBCA occurred at 0–3 months post-infusion in 11/22 (50%) and at 3–6 months in 11/22 (50%). Nine patients received upfront HSCT for early LBCA, two of these died of TRM. Eight patients received maintenance regime chemotherapy due to a contraindication to HSCT, unsuitable donors or family preference [20]. No non-relapse mortality (NRM) was observed in this group, 5 patients are alive in molecular remission (one after subsequent consolidative HSCT). Four patients received a repeat tisagenlecleucel infusion with lymphodepletion, none achieved long term B cell aplasia, 2 are alive in molecular remission (Fig. 4). We identified 4 patients that had early LBCA but did not receive any further treatment. All relapsed eventually 1, 1.5, 5.8 and 24.5 months after B-cell recovery; 1 died of disease, 1 is alive with disease awaiting HSCT, and 2 are alive after HSCT.

**Fig. 5:**
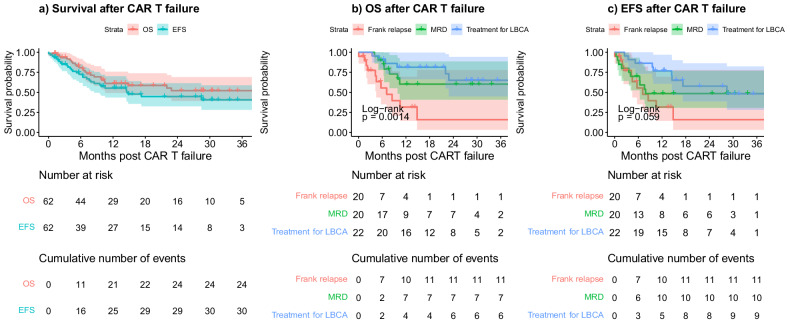
Survival after CAR T failure (*n* = 62). **a** OS and EFS for the 62 responding patients that subsequently failed CAR T. **b** OS and (**c**) EFS stratified according to type of CAR T failure. MRD measurable residual disease emergence, LBCA early loss of B-cell aplasia.

The OS and EFS 1 year after CAR T failure for these 62 patients were 61.2% (95%CI: 49.3–75.8) and 55.3% (95%CI 43.6–70.2), respectively. The median OS was not reached, the median EFS was 14.8 months (95%CI 8.5-NR) (Fig. 5a).

As expected, OS was significantly associated with the reason for tisagenlecleucel failure in this group (*p* = 0.0014). In other words, there was a worse outcome for patients who suffered frank relapse (1-year 31.7%, 95%CI 14.4–69.7%), compared to patients who had MRD emergence (1-year 60.2%, 95%CI 40.9–88.7%; HR 0.31, 95%CI 0.12–0.8) or who received further treatment for early LBCA (1-year 81.3%, 95%CI 66.4–99.7%; HR 0.20, 95%CI 0.07–0.56) (Fig. 5b).

Although the difference was not statistically significant for EFS (*p* = 0.06), EFS following treatment for early LBCA (1-year 76.8%, 95%CI 60.8–96.9; HR 0.35, 95%CI 0.14–0.86) and MRD emergence (1-year 48.5%, 95%CI 30.5–76.9; HR 0.58, 95%CI 0.24–1.39) was better than that following frank relapse (31.7%, 95%CI 14.4–69.7) (Fig. 5c). With a median follow-up of 21.5 months after CAR T failure (IQR 12.2–30.5), 28/62 (45.2%) maintained/attained a further molecular remission at the time of data cut-off. In all 49 patients with disease emergence after tisagenlecleucel, emergence of CD19 positive disease (*n* = 26) had similar outcomes when compared to CD19 negative disease (*n* = 15) (OS HR 0.68, 95%CI 0.27–1.72; EFS HR 0.84, 95%CI 0.38–1.88).

We analysed the effect of post-tisagenlecleucel HSCT on outcomes. 33/62 (53.2%) patients received HSCT at a median 14.8 months (95%CI 9.7–NR) after tisagenlecleucel infusion. For 6 of these 33 patients this was a second and for 1 it was the third transplant. 6/33 (18.2%) died of TRM (Supplementary Table 5), mainly due to infection. Only 1 of these 6 patients had had a prior HSCT. There was no NRM amongst the 29/62 patients who did not receive a transplant post-tisagenlecleucel failure.

A multivariable OS regression model including type of CAR T failure, prior HSCT, and HSCT after tisagenlecleucel (Table 6), confirmed the better outcome of patients who received further therapy for early LBCA compared to those with frank relapse (HR 0.24, 95%CI 0.1–0.7). Pre-CAR HSCT emerged as a risk factor for OS and EFS after CAR T failure (OS HR 2.53 95%CI 1.1–6.0, *p* = 0.03; EFS HR 2.3, 95%CI 1.1–4.9, *p* = 0.03) (Table 6a).

**Table 6:** Multivariable Cox regression model for survival after CART cell failure.

a.	OS			EFS		
Predictor variables	Hazard ratio	95%CI	*p*	Hazard ratio	95%CI	*p*
Post-CAR T HSCT*	0.94	0.4–2.4	0.89	1.09	0.5–2.6	0.85
Pre-CAR T HSCT	2.53	1.1–6.0	0.03	2.3	1.1–4.9	0.03
Type of CAR T failure						
Frank relapse	1			1		
MRD	0.39	0.1–1.1	0.02	0.74	0.3–1.8	0.16
Further treatment for early loss of B-cell aplasia	0.24	0.1–0.7		0.42	0.2–1.1	

b.	OS			EFS		
Predictor variables	Hazard ratio	95%CI	*p*	Hazard ratio	95%CI	*p*
Pre-CAR T HSCT	2.55	1.1–6.0	0.03	2.28	1.1–4.9	0.03
Type of CAR T failure						
Frank relapse	1					
MRD	0.39	0.1–1.1	0.02	0.75	0.3–1.9	0.15
Further treatment for early loss of B-cell aplasia	0.24	0.1–0.7		0.42	0.2–1.0	

*analysed as time-varying covariate.

Without post-CAR HSCT.

Models with (a) and without (b) post-CAR HSCT (as a time-varying covariate).

## Discussion

To the authors’ knowledge, this is the first truly population-based report on the outcomes of tisagenlecleucel for ALL. All patients deemed eligible for therapy at a periodic national meeting were included, giving a clinically-relevant intention-to-treat cohort including those for whom leukapheresis was not feasible. We applied a stringent definition of events which included all clinically-relevant circumstances including the need for further therapy for emergence of MRD or early LBCA as well as relapse or death from any cause [11, 21]. We also collected data on all but one infused patient even after failure of CAR T-cell therapy, to determine expected EFS and OS within this context. As such, we feel these outcome data provide clinicians with confidence to advise patients and their families on the complete CAR T pathway, including outcomes after this therapy fails.

In keeping with other paediatric real world data on CAR T therapy of ALL [6, 9, 10, 22], ours was a high-risk disease cohort, with over 40% transplanted patients and 38% patients proceeding to CAR T-cell therapy with ≥5% disease. The Paediatric Real World CAR Consortium (PRWCC) cohort reported real world outcomes on delivery of tisagenlecleucel for ALL in a US context. We found that 8/142 (5.6%) eligible patients were not harvested and out of those harvested 9/134 (6.7%) were not infused, compared with 15/200 (7.5%) in the PRWCC cohort [23]. Notably, progressive disease was found to be the cause in 8/17 (47%) non-infused patients in our cohort, highlighting the difficulty of bridging patients with highly aggressive disease. Nevertheless, the low drop-out rate demonstrates the feasibility of delivery of tisagenlecleucel on a national scale.

The EFS for our cohort (as defined in the ELIANA study, i.e. 46.5% at 2 years) reflects that found in other real-world studies [6, 9–11, 22]. However, this only partially informs outcomes for potential CAR T-cell therapy recipients, as a significant proportion of patients need further medical intervention after tisagenlecleucel due to MRD emergence and/or loss of B-cell aplasia/CAR T-cell persistence [24]. Thus, the stringent EFS better captures clinically-relevant outcomes necessitating further therapy after CAR T-cell infusion, with up to two thirds of patients requiring further intervention within 2 years from CAR T-cell infusion.

The CRS and ICANS incidence and severity presented here are comparable to those reported in similar cohorts. While most patients experienced CRS (55-68%) improved bridging strategies resulted in a lower incidence of severe CRS (16-20%) and ICANS (<10%); a single patient died of ICANS and none of CRS. Significant neutropenia in the post-CAR setting affected about 50% of patients but the rate of infectious complications was 28%, which is lower than reported in the literature [10, 15]. Hypogammaglobulinemia is a consequence of B-cell aplasia and affected most responding patients in this cohort.

The comprehensive follow-up on a national basis allowed robust reporting of complete post-infusion outcomes. Within responders, the 1-year EFS following CAR T-cell failure of 55.3% and OS of 61.2% are encouraging. A PRWCC analysis reported an OS of 52% after relapse, but notably this included patients with any level of disease that triggered new therapy. In contrast, our data newly demonstrate an improved outcome where further therapy is delivered on the basis of MRD emergence or early B-cell recovery. This may suggest there is a window for early intervention.

Reinfusion post lymphodepletion was ineffective in 4 patients and these findings are compatible with other studies [25, 26]. Interestingly, for a small group of patients that did not receive a HSCT as frontline treatment for early LBCA for several reasons, maintenance treatment emerged as a safe, low-cost alternative with equivalent outcomes to the HSCT patients. Additionally, whilst having had a prior HSCT did not affect outcomes for CAR therapy, it was significantly associated with worse outcomes after CAR T-cell failure. We noted a significant TRM (6/33, 18%) associated with delivery of post-CAR HSCT in our cohort of patients which is similar to that reported in other studies [7, 27].

The main limitations of this study are its retrospective nature and the relatively short follow-up following CAR T failure. However, the standardised and systematic data collection on a country-wide basis (only a single patient lost to follow-up) provides patients and clinicians with robust data to inform decision making around CAR T-cell treatment for r/r ALL. Our stringent EFS definition—as reported here in and in other cohorts [11, 28] - highlights a need to follow patients closely and to actively determine CAR T-cell failure at the earliest time-point, as this may allow a window for intervention with good short term outcome, even for patients with advanced disease. We suggest that going forward, stringent EFS is adopted as a clinically relevant and accurate outcome measure in CAR T studies.

### Supplementary information

Supplemental material

## Data Availability

The deidentified datasets generated during and/or analysed during the current study are available from the corresponding author on reasonable request.
