# Supplementary material for: Intention-to-treat outcomes utilising a stringent event definition in children and young people treated with tisagenlecleucel for r/r ALL through a national access scheme
Source: Blood Cancer J. 2024 Apr 15;14(1):66. doi: 10.1038/s41408-024-01038-2 (PMC11018620; doi:10.1038/s41408-024-01038-2)
Supplement: Supplementary file 1 — Supplemental material [file 41408_2024_1038_MOESM1_ESM.pdf]

Supplementary material for manuscript "Intention-to-treat outcomes utilising a stringent event definition in children and young people treated with tisagenlecleucel for r/r ALL through a national access scheme", Oporto M et al. 2024.

- Supplementary Table 1: Sites of non-CNS extramedullary disease prior to tisagenlecleucel
- Supplementary Table 2. KMT2A rearrangements (n=21)
- Supplementary Table 3. Details of manufacture failure patients
- Supplementary Figure 1. Impact of baseline characteristics on EFS (as per ELIANA criteria).
- Supplementary Table 4. Viral infection details
- Supplementary Table 5. Causes of transplant related mortality (TRM) after post-tisagenlecleucel HSCT.

Supplementary Table 1. Sites of non-CNS extramedullary disease prior to tisagenlecleucel (n=17 patients, some with multiple sites involved)

7 Testicular
1 Liver lesions
1 Parietal and extraosseous
2 Lymph nodes
1 Skin and muscle infiltration
1 Epidural
1 Dural (parietal)
2 Ocular
1 Parotid, scalp and breast
1 Retroorbital and left renal mass
1 Paravertebral mass

Supplementary Table 2. KMT2A rearrangements (n=21)

	n
t(1;11)	1
t(4;11)	4
t(9;11)	2
t(11;19)	3
3' deletion of KMT2A	1
Other/not available	10

Supplementary Table 3. Details of manufacture failure patients

CR = complete remission, DoD = died of disease, LBA = loss of B-cell aplasia, OOS = out of specification infusion

Patient	Manufacture failure	Action	Outcome
MF1	Failed, <70% Viability	OOS infusion	Infused, no toxicity, CR at day 30, DoD
MF2	Failed	Progressed, couldn't reattempt apheresis	DoD
MF3	Failed, cause unknown	Successful repeat harvest and manufacture, infused	Alive after HSCT for early LBA
MF4	Failed	Successful repeat harvest and manufacture, infused	DoD prior to day 30
MF5	Failed, <2.2% transduction efficiency	Successful repeat harvest and manufacture, not infused	Died in remission of infection after lympho-depletion, background of severe renal impairment

Supplementary Figure 1. Impact of baseline characteristics on EFS (as per ELIANA criteria). a) BM disease burden prior to lymphodepletion b) Blinatumomab response c) Refractory disease at any point before tisagenlecleucel d) Inotuzumab exposure and e) Refractory relapse. p values are for Log-rank comparison of curves.

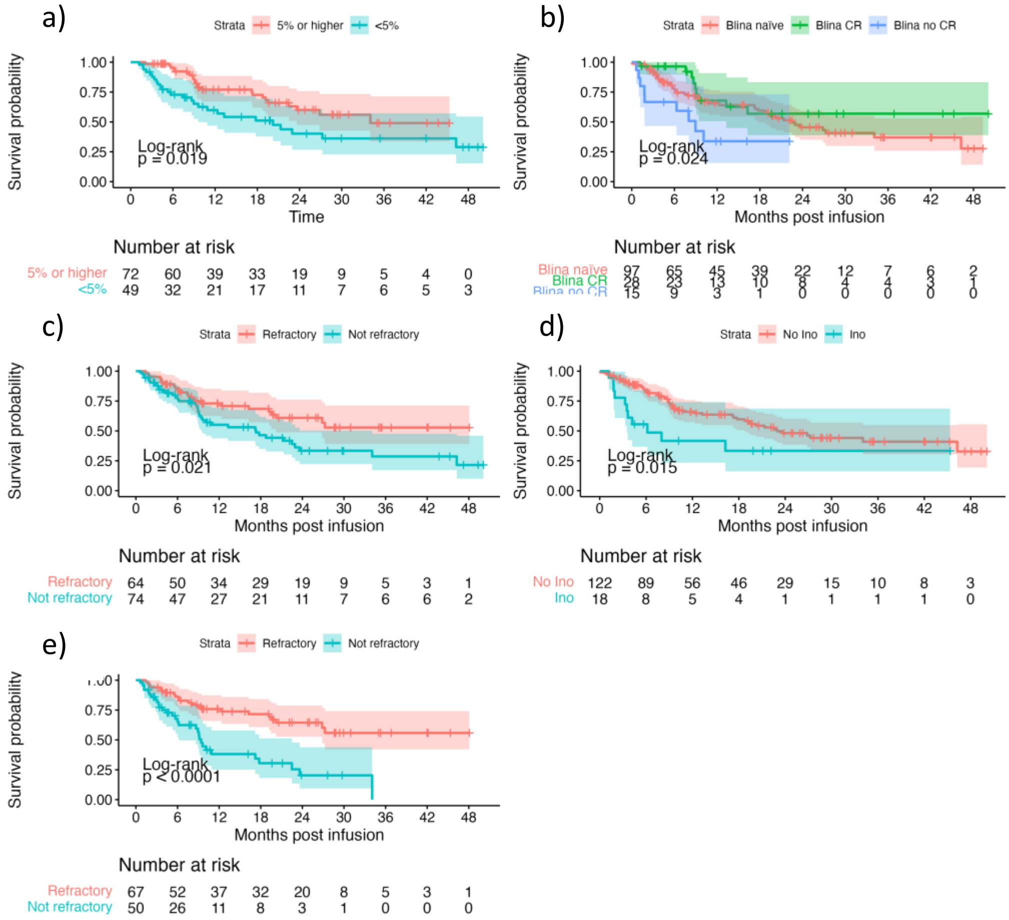

Supplementary Table 4. Viral infection details

Viral infection (n=10)	n
BK virus	1
Parvovirus	1
Rhinovirus	2
Covid-19	2
Adenovirus	1
JC and HHV6	1
Influenza	1
Adeno+Covid-19	1

Supplementary Table 5. Causes of transplant related mortality (TRM) after post-tisagenlecleucel HSCT.

n=6		
ID	Cause of TRM	Days from post-CAR HSCT until death
TRM1	Neutropenic sepsis and multiorgan failure	13
TRM2	Thrombotic microangiopathy and HSV infection witl	53
TRM3	Adenovirus pneumonitis	61
TRM4	VOD/Adenoviraemia	80
TRM5	Perianal sepsis following cord graft rejection	156
TRM6	Secondary graft failure, followed by second HSCT (haplo) and invasive fungal respiratory infection	274
